# Associations of C-reactive protein with depressive symptoms over time after mild to moderate ischemic stroke in the PROSCIS-B cohort

**DOI:** 10.1007/s00415-023-12038-w

**Published:** 2023-10-18

**Authors:** Viktoria L. K. Schaeff, Pia S. Sperber, Sophie K. Piper, Naomi K. Giesers, Karen Gertz, Peter U. Heuschmann, Matthias Endres, Thomas G. Liman

**Affiliations:** 1 Charité—Universitätsmedizin Berlin, Center for Stroke Research Berlin, Charitéplatz 1, 10117 Berlin, Germany; 2 German Centre for Cardiovascular Research DZHK, Berlin, Berlin, Germany; 3 Charité—Universitätsmedizin Berlin, Department of Neurology With Experimental Neurology, Berlin, Germany; 4 Charité—Universitätsmedizin Berlin, Experimental and Clinical Research Center, Berlin, Germany; 5 Charité—Universitätsmedizin Berlin, Institute of Biometry and Clinical Epidemiology, Berlin, Germany; 6 Berlin Institute of Health, Berlin, Germany; 7 Charité—Universitätsmedizin Berlin, Institute of Medical Informatics, Berlin, Germany; 8 Department of Neurology, Carl Von Ossietzky University, Oldenburg, Germany; 9 Institute of Clinical Epidemiology and Biometry, University of Würzburg, Würzburg, Germany; 10 Clinical Trial Center Würzburg, University Hospital Würzburg, Würzburg, Germany; 11 Institute for Medical Data Science, University Hospital Würzburg, Würzburg, Germany; 12 German Center for Neurodegenerative Disease DZNE, Berlin, Germany; 13 Charité – Universitätsmedizin Berlin, Neurocure Cluster of Excellence, Berlin, Germany

**Keywords:** Inflammation, Hs-CRP, Ischemic stroke, Depression

## Abstract

**Background and purpose:**

C-reactive protein serves as a marker of inflammation and is linked to depression in the general population. We aimed to assess whether elevated baseline levels of high-sensitivity C-reactive protein (hs-CRP) are associated with depressive symptoms over time in a prospective cohort of mild-to-moderate first-ever ischemic stroke patients.

**Methods:**

Data were obtained from the Prospective Cohort with Incident Stroke Berlin (NCT01363856). Depressive symptoms were assessed with the Center for Epidemiologic Studies Depression Scale (CES-D) at three annual follow-up points. We assessed the association of elevated levels of hs-CRP with CES-D scores over time via linear mixed models. In a subgroup analysis, we explored an interaction effect with sex.

**Results:**

We included 585 ischemic stroke patients with baseline data on CRP levels. The mean age was 67 (13 SD), 39% (*n* = 226) were female, and the median National Institutes of Health Stroke Scale (NIHSS) was 3 (IQR 1–4). Twenty percent of survivors showed evidence for depressive symptoms one year after stroke with CES-D ≥ 16, 21% at year two, and 17% at year three. Higher log-transformed baseline hs-CRP levels were associated with higher CES-D Scores over time in the adjusted linear mixed model (*β* = 1.28; (95% CI 0.22–2.34)). The subgroup analysis revealed an interaction effect of hs-CRP on depressive symptoms in women (*β* = 2.33; (95% CI 0.71–3.95)).

**Conclusion:**

In our cohort with mild-to-moderate first-ever ischemic stroke patients, hs-CRP levels were associated with more depressive symptoms over time, with an interaction effect for the female sex.

**Study registration:**

https://clinicaltrials.gov; Unique identifier: NCT01363856.

**Supplementary Information:**

The online version contains supplementary material available at 10.1007/s00415-023-12038-w.

## Introduction

Depression is a major complication after stroke affecting more than 30% of patients up to five years after stroke [1]. Depressive symptoms after stroke, especially PSD (post-stroke depression), are associated with poor outcomes including increased mortality and disability [2]. Inflammation has been linked to both risk of cardiovascular events and depression [3]. Recent studies in the general population reported an inflammatory response contributing to the development of major depressive disorders via biological mechanisms such as altered neurotransmitter metabolism, increased proinflammatory cytokines, and glucocorticoid receptor resistance [4, 5]. However, the role of inflammatory processes in the pathophysiology of depressive symptoms after stroke is less apparent [6, 7]. Some studies have shown an association of increased peripheral inflammation in PSD [8, 9], while in another analysis this association was lost over time [10]. Additionally, some studies have only reported the presence of low-grade peripheral inflammation without significant association with PSD [11].

However, the natural course of depressive symptoms after ischemic stroke is variable, and relying on single measures of depressive symptoms might limit research on a potential association between inflammation and depressive symptoms after stroke.

Here, we aimed to assess whether elevated baseline levels of high-sensitivity C-reactive protein (hs-CRP) are associated with depressive symptoms over time in a prospective cohort with annual assessments of depressive symptoms via CES-D (Center for Epidemiologic Studies Depression Scale) in ischemic stroke patients.

## Methods

### Data availability

The data and syntax supporting our findings are available from the principal investigator (Dr Liman) upon reasonable request.

### Study design and patient characteristics

The PROSCIS-B (Prospective Cohort with Incident Stroke—Berlin, *ClinicalTrials.gov identifier: NCT01363856*) is a prospective, observational hospital-based cohort study that recruited adult patients at three tertiary university hospital sites of the Charité-Universitätsmedizin Berlin with first-ever stroke according to WHO criteria [12]. Patients diagnosed with prior stroke, brain tumor, or brain metastasis of other origin, or patients participating in an interventional study were excluded. We only included patients presenting without moderate to severe aphasia or dysarthria due to ethical regulations. Details on study design, in-, and exclusion criteria have been published previously [13, 14]. For this study, only patients with mild-to-moderate ischemic stroke events (NIHSS (National Institutes of Health Stroke Scale) < 16) were included, as cases with severe strokes were few (NIHSS $\ge$ 16, *n* = 6). A detailed baseline characterization of patients with extensive clinical and technical examinations was performed within 7 days after symptom onset including blood sampling for laboratory measures as previously described [13].

Patients were contacted annually to assess depressive symptoms, cognitive function, and functional outcomes up to three years after the index event.

Additionally, magnetic resonance imaging (MRI) data were gathered using different 1.5-T and 3-T scanners at three tertiary sites. Fluid-attenuated inversion recovery or T2-weighted images were used to assess white matter lesion severity according to the Wahlund classification system [15]. The Wahlund visual rating scale, or age-related white matter changes scale (ARWMC), is a scale ranging from a score of 0 to 30 that accounts for white matter lesions (WML) in both sides of the brain (right and left) and pre-specified brain regions (frontal, parieto-occipital, temporal, infratentorial/cerebellum, and basal ganglia). Here, white matter hyperintensities are assigned a score of 0 to 3 in each region on both sides of the brain. The final score is the sum of all regions. The rating was performed independently by two evaluators.

### CRP measurements

Blood serum samples were obtained within seven days after the index event. Chemiluminescent immunoassay (Siemens) was used to determine the concentration of hs-CRP. Detection limits ranged from 0.3–101 mg/L with an analytical sensitivity of 0.1 mg/L and a functional sensitivity of 0.3 mg/L.

### Outcome definitions

The outcome of interest was depressive symptoms after stroke. Symptoms of depression were assessed annually until three years after the stroke with a CES-D score (20 items, 4 domains, range 0–60) by postal mail or telephone interview. CES-D Scores ≥ 16 are defined to be indicative of the presence of Depression [16]. Patients without a CES-D assessment available at any of the three follow-up points, e.g., due to death, have also been excluded from our analysis. Verification of death was obtained by the Berlin registry office.

### Statistical methods

Baseline descriptive statistics are given as mean with standard deviation (SD) and median with limits of the interquartile range (IQR). The internal consistency of responses was evaluated by Cochrane’s alpha. Moreover, we performed linear mixed models (LMM) to calculate crude and confounder adjusted effect sizes (*β*) and corresponding 95% Confidence Intervals (CI) of baseline hs-CRP on CES-D Score for the period of three years after the first ischemic stroke. LMM tolerates missing data and accounts for repeated measurements in subjects by fitting a random intercept for each patient. Because the hs-CRP distribution was right skewed, we log (10*X*_i_ + 0.01) transformed hs-CRP. Due to the log transformation, β accounts for the increase in depressive symptoms (CES-D Score) for each increase of hs-CRP by a factor of 10 on a multiplicative scale. Dose–response relationship of hs-CRP on CES-D Score over time was investigated by dividing hs-CRP into quartiles and comparing the effect size (*β*; 95% CI) to the first quartile using LMM. Exclusively for investigating dose response, we did not log transform hs-CRP to keep the quartiles more comprehensible.

Confounders for our adjusted model were systematically identified by creating a DAG (directed acyclic graph) [17]. A causal diagram for variable selection was drafted with DAGitty (http://www.dagitty.net/dags.html, see Supplemental Fig. 1). Variables included as confounders in the final model were age (continuous), sex (female/male), diabetes mellitus (dichotomous), history of cardiovascular disease (dichotomous), NIHSS (continuous), BMI (continuous), current smoker (dichotomous), habitual alcohol consumption (dichotomous), and activity level pre-stroke (dichotomous; none to occasional/ regular to heavy).

We identified white matter lesions as a marker for cerebral small vessel disease, which was considered a possible confounder in the DAG, but MRI data were limited to *n* = 402 patients. Therefore, we ran a separate analysis including only those patients with MRI data available in which the Wahlund Score was added as a confounder in the multivariable analysis. Additionally, we investigated whether the effect of hs-CRP on more depressive symptoms depends on sex. For this, we repeated our main analysis by adding an interaction term of sex and hs-CRP.

In a later added secondary analysis, we have identified individuals taking antidepressant or anti-inflammatory medication and repeated our multivariate analysis including those as confounders. Antidepressant and anti-inflammatory medication use (dichotomous) was established via anatomical-therapeutic-chemical Classification (ATC) Codes 2018 at admission (antidepressant medication: N06A and N06C and anti-inflammatory medication: H02A-B, J01A-X, J05A, M01A-C).

Data preparation was done using IBM SPSS Statistics for Windows (v.24, IBM Corp, Armonk, NY). All descriptive and statistical analyses were performed in R (4.2.2.) with the RStudio user interface. We adhered to STROBE (Strengthening the Reporting of Observational Studies in Epidemiology) criteria.

### Ethical approval

Patients or their legal representatives gave written consent. The PROSCIS-B study was approved by the Ethics Committee of the Charité—Universitätsmedizin Berlin (EA1/218/09) and was conducted according to the ethical principles stated by the Declaration of Helsinki.

## Results

### Baseline data

**Fig. 1:**
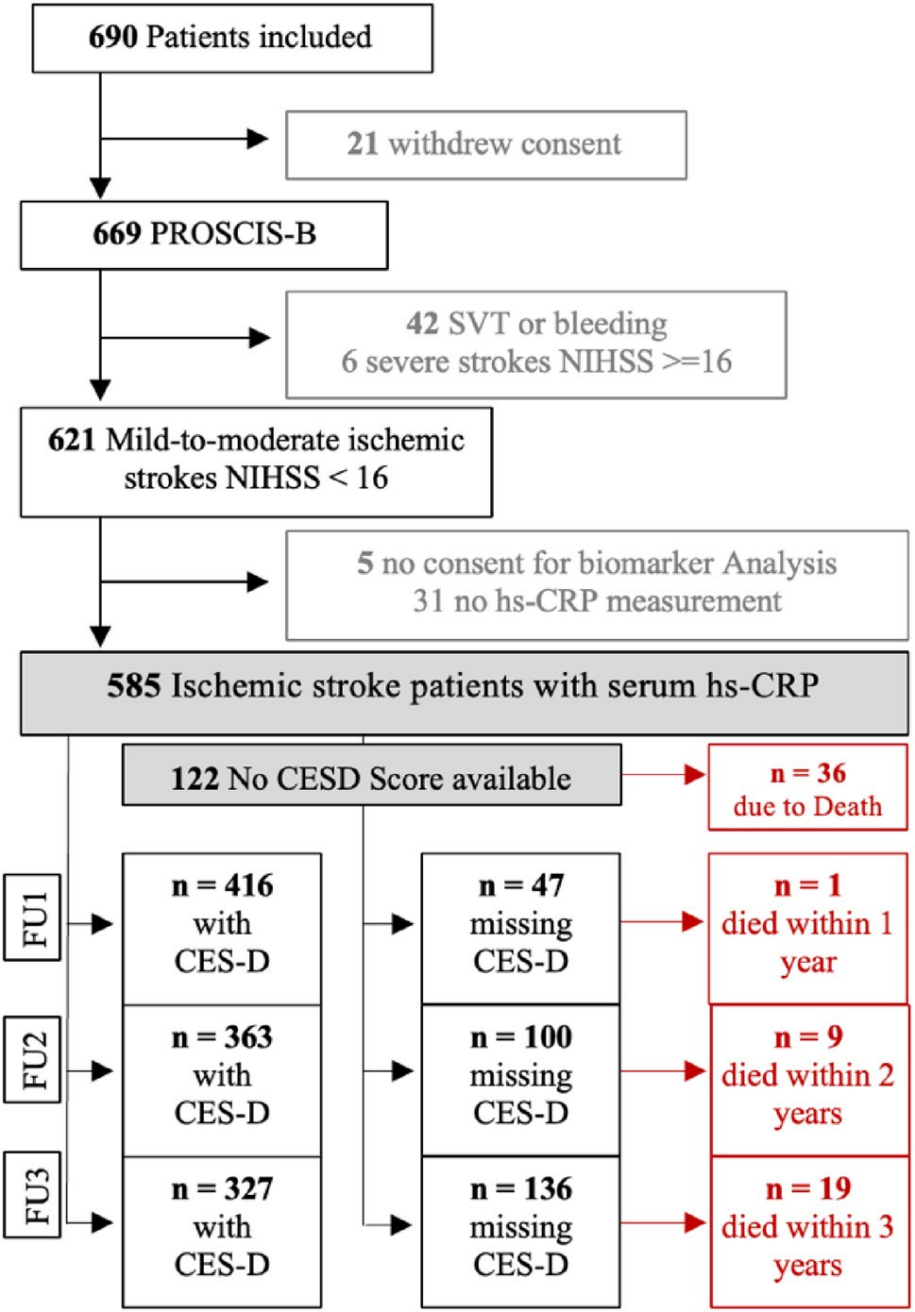
Flowchart of inclusion and exclusion criteria, consent included consent for study participation and biomarker analysis, *PROSCIS-B* Prospective Cohort with Incident Stroke Berlin, *NIHSS* National Institutes of Health Stroke Scale, *hs-CRP* high-sensitivity C-reactive Protein, *CES-D* Center for Epidemiologic Studies Depression Scale

We included 621 patients with mild to moderate stroke between March 2010 and May 2013 in the PROSCIS B study. PROSCIS-B patients had a mean age of 67 (± 13SD) years, 39% (*n* = 242) were female, and the median baseline NIHSS score was 2 (IQR 1–4). We obtained hs-CRP levels in 585 ischemic stroke patients. In our longitudinal analysis, we included 463 patients who additionally provided at least one CES-D assessment during follow-up. Details on the number of patient inclusion and exclusion are presented in the flowchart in Fig. 1. Baseline characteristics of PROSCIS-B participants are presented in detail in Table 1**.** The median time for retrieving blood samples to determine hs-CRP was 4 days (IQR 3–5) and did not significantly differ in patients with elevated (≥ 5 mg/L) versus normal hs-CRP values. Hs-CRP ranged from 0.3 to 101 mg/L in the analyzed sample. In 55% of patients, we received complete CES-D Data on all three follow-up points. Cochranes’s alpha for CES-D was 0.9 for all three assessments.

**Table 1:** Demographic baseline characteristics stratified by high sensitivity C-reactive protein (hs-CRP) levels

Variable	hs-CRP < 5 mg/L *n* = 301	hs-CRP ≥ 5 mg/L, *n* = 284	*p*-value^1^
Age in years mean (SD)	66 (13)	71 (13)	< 0.001
Female sex *n* (%)	102 (34%)	124 (44%)	0.015
Diastolic blood pressure mmHg, mean (SD)	78 (13)	75 (16)	0.013
Systolic blood pressure mmHg, mean (SD)	139 (22)	137 (22)	> 0.9
NIHSS Score, median (IQR)	2.0 (1.0, 4.0)	3.0 (1.0, 5.0)	< 0.001
0–4	245 (81%)	196 (69%)	
5–15	56 (19%)	88 (31%)	
≥ 16	0 (0%)	0 (0%)	
Habitual alcohol consumption *n* (%)*	115 (40%)	88 (32%)	0.043
Current smoker, *n* (%)*	88 (30%)	75 (27%)	0.4
BMI in kg, median (IQR), *n* (%)*	26.3 (23.8, 28.9)	27.5 (24.7, 30.9)	< 0.001
< 18.5	2 (0.7%)	2 (0.7%)	
≥ 18.5 and < 25	122 (41%)	78 (28%)	
≥ 25 and < 30 t	121 (40%)	114 (41%)	
≥ 30	55 (18%)	84 (30%)	
Total cholesterol (mg/dL), mean (SD)	198 (44)	191 (53)	0.12
High-density lipoprotein (mg/dl), mean (SD)	50 (37)	47 (44)	< 0.001
Low-density lipoprotein (mg/dl), mean (SD)	121 (16)	114 (16)	0.029
Triglyceride (mg/dl), mean (SD)	116 (73)	120 (87)	0.4
Diabetes mellitus, *n* (%)	60 (20%)	68 (24%)	0.2
History of myocardial infarction, *n* (%)	10 (3.3%)	11 (3.9%)	0.7
Coronary artery disease, *n* (%)	47 (16%)	44 (15%)	> 0.9
Peripheral artery disease, *n* (%)	16 (5.3%)	24 (8.5%)	0.13
Arterial hypertension, *n* (%)	176 (58%)	207 (73%)	< 0.001
Atrial fibrillation, *n* (%)	41 (14%)	83 (29%)	< 0.001
Stroke subtype according to TOAST, *n* (%)			0.042
Large artery atherosclerosis	74 (25%)	80 (28%)	
Cardioembolic	60 (20%)	79 (28%)	
Small artery occlusion	51 (17%)	43 (15%)	
Other cause	12 (4.0%)	5 (1.8%)	
Undefined	104 (35%)	77 (27%)	
Pre-stroke Institutionalization, *n* (%)	5 (1.7%)	6 (2.1%)	0.7
Education, *n* (%)*			< 0.001
≤ 10 years	186 (64%)	211 (79%)	
> 10 years	105 (36%)	56 (21%)	
Physical activity pre-stroke, *n* (%)*			< 0.001
None to occasional	174 (58%)	218 (78%)	
Regular to heavy	125 (42%)	62 (22%)	
WahlundScore, median (IQR) ^#^	5.0 (2.0, 8.0)	6.0 (3.0, 11.0)	0.004
Infarct pattern, *n* (%) ^#^			0.8
Territorial with subcortical and cortical	63 (30%)	52 (32%)	
Subcortical	60 (29%)	40 (25%)	
Scattered infarct	45 (22%)	38 (23%)	
Lacunar	0 (0%)	1 (0.6%)	
Infratentorial	41 (20%)	31 (19%)	

1 Wilcoxon rank sum test; Pearson's Chi-squared test; Fisher's exact test

^*^Restricted to patients without missing values in the respective category

^#^MRI available in *n* = 402 patients

In our cohort, 35 patients received antidepressant medication at baseline. However, 10 individuals had to be excluded from the analysis as they did not provide a single CESD Score. Among these individuals (52% (*n* = 13) were female), 13 used tricyclic antidepressants, seven were on selective serotonin reuptake inhibitors, three used serotonin and norepinephrine reuptake inhibitors, and three took tetracyclic antidepressants, while one used a double combination. At first-year follow-up, 20% (*n* = 86) were depressed, indicated by a CES-D score ≥ 16 and 47% (*n* = 8) at year two, and 50% (*n* = 9) at year three.

### Follow-up data

The median follow-up time for the third appointment was 1125 days (IQR 1093 – 1213). Median CES-D score at first-year follow-up was 7 (IQR 3–14) for patients with hs-CRP levels below the clinical threshold (< 5 mg/L), while patients with elevated hs-CRP levels (≥ 5 mg/L) presented with a median CES-D score of 9 (IQR 4–14). At first-year follow-up, 20% (*n* = 86) were depressed, indicated by a CES-D score ≥ 16. It was 21% (*n* = 77) in year two and 17% (*n* = 56) in year three. A detailed overview of the cohort at each follow-up point is presented in Supplemental Table 1. In summary, patients with a CES-D score of ≥ 16 were more often female, presented with higher NIHSS Score, received < 10 years of education, and had a history of coronary artery disease.

In the longitudinal (repeated measures) analysis, higher baseline hs-CRP levels were associated with higher CES-D Scores over the period of three years in unadjusted linear mixed models with a crude *β* = 1.87; (95% CI, 0.88–2.86) and in the adjusted models with *β* = 1.28; (95% CI 0.22–2.34). Furthermore, we found an interaction for women in hs-CRP on CES-D scores over time *β* = 2.33; (95% CI 0.71–3.95), as shown in Table 2.

**Table 2:** High sensitivity C-reactive protein and depressive symptoms over time after stroke

	Crude		Adjusted		Adjusted + male interaction		Adjusted + female interaction	
	Change in CES-D score over time							
	Beta	95% CI	Beta	95%- CI	Beta	95% CI	Beta	95% CI
10log hs-CRP	1.87***	0.88–2.86	1.28*	0.22 –2.34	0.61	− 0.71 –1.93	2.33**	0.71 –3.95

^#^Adjusted for age, sex, physical activity pre-stroke, diabetes mellitus, history of cardiovascular disease, stroke severity, BMI, regular alcohol consumption, and smoking status; *p < 0.05, **p < 0.01, ***p < 0.001

### Sensitivity analyses

Additionally, we performed a sensitivity analysis in stroke patients with baseline data on MRI images adding the Wahlund visual rating scale, or ARWMC as a confounder in our linear mixed model as shown in Table 3. WML are areas of abnormal myelination in the brain that reflect e.g., inflammation and damage to the myelin and are considered to be a valid marker for small vessel disease [18]. Higher baseline hs-CRP levels were also associated with higher CES-D Scores over the period of three years in the unadjusted linear mixed models with a crude *β* = 1.67; (95% CI, 0.53–2.81) and after full adjustment *β* = 1.58; (95% CI, 0.36–2.8) in 328 stroke patients with data on MRI. Once more, we identified an interaction for the female sex of hs-CRP on CES-D scores over time shown by *β* = 2.82; (95% CI 0.93–4.70).

**Table 3:** High sensitivity C-reactive protein and depressive symptoms over time after stroke adjusted for cerebral white matter hyperintensities in patients with data on baseline MRI

	Crude		Adjusted		Adjusted + male interaction		Adjusted + female interaction	
	Change in CES-D score over time							
	Beta	95% CI	Beta	95%- CI	Beta	95% CI	Beta	95% CI
10log hs-CRP	1.67**	0.53–2.81	1.58*	0.36 –2.8	0.79	− 0.73 –2.31	2.82**	0.93 –4.70

^#^Adjusted for age, sex, physical activity pre-stroke, ARWMC (Wahlund Score), diabetes mellitus, history of cardiovascular disease, stroke severity, BMI, regular alcohol consumption, and smoking status; *p < 0.05, **p < 0.01, ***p < 0.001

The results of the dose–response analysis are shown in Supplemental Table 2. In summary, we observed a slight dose–response of hs-CRP levels in the quartiles of CES-D over time. The highest levels of hs-CRP (12.5–101 mg/L, quartile 4) were statistically significantly associated with higher CES-D scores over time in the adjusted model *β* = 1.96; (95% CI 0.02–3.91). However, in the interaction analysis for women, we identified hs-CRP levels of 4.77–12.5 mg/L (quartile 3) to be statistically significantly associated with higher CES-D score over time in the adjusted model *β* = 3.87; (95% CI 0.76–6.99).

We conducted a final subgroup analysis in stroke patients with hs-CRP levels below 10 mg/L indicating low-grade inflammation. Hs-CRP levels over 10 mg/L have been described as a cut-off for clinically significant inflammation [19]. Results are shown in Supplemental Table 3. In stroke patients with hs-CRP < 10 mg/L (*n* = 331), higher hs-CRP levels over a 3-year period were not associated with higher CES-D scores. However, when introducing an interaction term for the female sex, we observed an association: *β* = 3.54; (95% CI 0.92–6.2).

In sensitivity analyses adjusted for anti-inflammatory and antidepressant medication, no major differences were found (adjusted: *β* = 1.26; (95% CI 0.21–2.32), female *β* = 2.27; (95% CI 0.67–3.88), male *β* = 0.63; (95% CI − 0.67–1.93)).

## Discussion

In our study of mild-to-moderately affected first ischemic stroke patients, we demonstrated that higher baseline hs-CRP levels were associated with more depressive symptoms during a follow-up of three years. In subgroup analysis, after adjustment for WML, the effect size increased. Consistently, our results showed an interaction with the female sex. Additionally, we found a slight dose–response relationship between hs-CRP levels and depressive symptoms over time, although the effect was more pronounced in women with “low-grade inflammation” (hs-CRP < 10 mg/L).

Several observational studies have reported associations of inflammatory biomarkers such as CRP, TNF-α, and Interleukin-6 (IL-6) with PSD/depressive symptoms after stroke [7, 8, 20]. In a larger prospective stroke cohort, Kowalska et al. showed that elevated levels of CRP measured within 48 h after stroke onset were associated with depressive symptoms at day 8, but not with depressive symptoms after stroke three months later [9]. Others reported higher levels of CRP and leukocytes present at 18 months after stroke but did not find an association between elevated CRP levels and PSD [10]. A recent systematic review with data from 3,536 stroke patients from 13 cohort studies (12 Chinese, 1 European) demonstrated that higher CRP levels in the acute phase of stroke indicate a higher risk for PSD during follow-up of one year [20]. The review specifically called for further studies with extended follow-up periods as the majority (ten) of studies only provided a follow-up time of three months. They concluded, primarily based on short follow-up times, that elevated CRP might serve as a predictor for PSD. However, further observational studies with longer follow-up times are required. They found an overall prevalence of PSD of approximately 30%, and patients with PSD had significantly higher CRP levels on admission compared to non-PSD patients (standard mean difference: 0.19; 95% CI 0.12–0.27). In our study, the prevalence of depression was 20% at year one, 21% at year two, and 17% at year three. Thus, the prevalence was comparable and consistent with reports from other hospital cohorts in which the incidence of PSD ranged between 11 and 30% up to four years after stroke [1].

However, significant variability between studies was found, resulting from heterogeneity in clinical, methodological, and statistical factors (e.g., follow-up time points, inclusion criteria, assessment of depressive symptoms, and definition of PSD/depression after stroke) For instance, in the systematic review the timing of PSD assessment varied between 8 days and 12 months after stroke [20].

Consistent with these findings, our study demonstrated, for the first time, that the previously described effect of CRP on depressive symptoms continues to persist up to three years after stroke. In a sub-study of the population-based Rotterdam study, elevated IL-6 and CRP levels were found to independently predict incident depressive symptoms (CES-D $\ge$ 16) over five years, irrespective of age, gender, BMI, physical illness, smoking status, or MMSE. Moreover, higher baseline levels of IL-6 and CRP were also associated with the persistence of depressive symptoms over five years, whereas none of the inflammatory markers were associated with depressive symptoms at baseline [21].

Several mechanisms have been proposed as a possible link between inflammation and depression [22–25]. This so-called “inflammation hypothesis” of depression has also been applied to PSD [6]. One explanation might be a reduced function of the hypothalamus–pituitary–adrenal axis in patients with major depression and chronic inflammation [26]. Elevated levels of inflammatory mediators could lead to a reduction in brain-derived neurotrophic factor, which negatively influences neurogenesis and neuroplasticity [27] and is also associated with depression [28]. Further evidence supporting the hypothesis that inflammation is a cause rather than a consequence of depression is provided by a Mendelian randomization analysis of a UK Biobank sample, demonstrating that IL-6 and CRP are causally linked with depression [29].

In sensitivity analyses (interaction analyses), the association between hs-CRP and depressive symptoms over time was more pronounced in women, especially in those with low-grade inflammation. A recent study found a strong association between CRP and depression in women that aligns with our results [30]. The lifetime risk for depression in women is twice as high as in men, showing consistent patterns across cultures [31]. According to a recent review, the association between inflammation and depression may be particularly relevant in women [32]. For instance, more women tend to have elevated inflammatory markers such as CRP compared to men [33]. Additionally, healthy women with higher baseline levels of CRP had more depressive symptoms over a 7-year follow-up period than those with lower values [34]. Several factors that promote inflammation, including distress syndrome, childhood adversity, and obesity appear to affect women to a greater extent than men, which may help to explain why the effect of elevated hs-CRP levels on depressive symptoms is pronounced in women in our study [35].

There are several strengths and limitations of our study to be considered. The strength of our study lies in the prospective design, extended follow-up period, and the systematic identification of confounders.

However, there are limitations that should be acknowledged. First, we only assessed baseline hs-CRP levels in the acute phase of stroke. Repeated measures of proinflammatory markers (e.g., hs-CRP and IL-6) over time would have provided more insights into the temporal relationship between inflammation and depression. Second, it is essential to acknowledge a limitation related to the assessment of psychiatric status. Depressive symptoms were evaluated solely through a self-reported 20-item questionnaire and was not confirmed by a clinical psychiatric assessment. Additionally, the omission of participants’ prior psychiatric history represents a potential limitation, given that our analysis relied exclusively on baseline antidepressant medication usage. In the same context, the ongoing monitoring of antidepressant medication utilization during follow-up periods could yield valuable insights into clinically relevant depressive episodes and serve as a covariate for future investigations.

In addition, in this study, we were not able to adjust for clinical infection in the post-stroke phase or during follow-up. This might introduce a bias in our results and should be considered when interpreting our findings, while we assume that infections would be not favored in one sex. However, in the subgroup analysis for low-grade inflammation, we confirm our findings.

Fourth, our findings may only be applicable to mild and moderate stroke patients, as severe stroke was an exclusion criterion. Additionally, during all follow-up points, we had a considerable number of patients who did not report back. However, the linear mixed-effect model is well known for its ability to handle missing data [36].

Lastly, the aim of this study was to investigate high-sensitivity C-reactive protein as a potential contributing factor to post-stroke depressive symptoms. Depression is a complex condition influenced by a variety of genetic, environmental, and physiological factors, with hs-CRP being just one of many potential contributors. Therefore, this is crucial when interpreting these findings.

## Conclusion

In our cohort with mild-to-moderate first-ever ischemic stroke patients, higher levels of baseline hs-CRP were associated with more depressive symptoms over time. This effect was pronounced in women, particularly in women with hs-CRP values indicating low-grade inflammation. Further longitudinal studies (including cytokine analysis such as IL-6) are warranted to investigate the temporal relationship between sex, inflammation, and depressive symptoms after stroke.

### Supplementary Information

Below is the link to the electronic supplementary material.

Supplementary file1 (DOCX 383 KB)
