# Supplementary material for: Associations of C-reactive protein with depressive symptoms over time after mild to moderate ischemic stroke in the PROSCIS-B cohort
Source: J Neurol. 2023 Oct 18;271(2):909–17. doi: 10.1007/s00415-023-12038-w (PMC10828033; doi:10.1007/s00415-023-12038-w)
Supplement: Supplementary file 1 — Supplementary file1 (DOCX 383 KB) [file 415_2023_12038_MOESM1_ESM.docx]

**Supplement**

**Figure legends:**

Supplemental figure 1. Directed anticyclic graphs for Confounder identification of total effect of high sensitivity C-reactive protein on depressive symptoms using the Center for Epidemiologic Studies Depression Scale


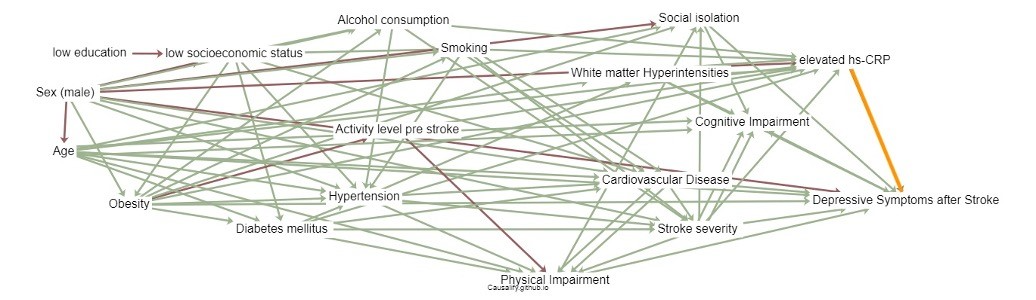


Supplemental Table 1. – Patient characteristics stratified for follow-up point and depression defined as CES-D ≥ 16

|  | Follow-up Year 1 | | | | Follow-up Year 2 | | | | Follow-up Year 3 | | | |
| --- | --- | --- | --- | --- | --- | --- | --- | --- | --- | --- | --- | --- |
| CES-D Score < 16, n  CES-D Score $\geq$ 16, n (%)  No CES-D Score available, n  No CES-D Score due to Death, n (%)  Median CES-D (IQR) | 330 (80 %)  86 (20%)  169  37 (21 %)  7 (3 – 14) | | | | 286 (79 %)  77 (21 %)  222  45 (20 %)  7 (3 – 15) | | | | **271 (83 %)**  56 (17 %)  258  55 (21 %)  7 (3 – 14) | | | |
| **Variable** | **N** | CES-D < 16 | CES-D ≥ 16 | **p-value^1^** | **N** | CES-D < 16 | CES-D ≥ 16 | **p-value^1^** | **N** | CES-D < 16 | CES-D ≥ 16 | **p-value^1^** |
| Age in years, mean (SD) | 416 | 66 (13) | 65 (12) | 0.2 | 363 | 66 (12) | 63 (13) | 0.024 | 327 | 65 (12) | 68 (11) | 0.2 |
| Female sex, n (%) | 416 | 115 (35%) | 46 (53%) | 0.002 | 363 | 97 (34%) | 35 (45%) | 0.062 | 327 | 88 (32%) | 28 (50%) | 0.013 |
| hs-CRP in mg/L^1^ | 416 | 4 (2, 11) | 5 (2, 12) | 0.15 | 363 | 4 (2, 10) | 6 (2, 15) | 0.021 | 327 | 3 (1, 8) | 6 (3, 19) | <0.001 |
| NIHSS Score, n (%) | 416 |  |  | 0.3 | 363 |  |  | 0.2 | 327 |  |  | 0.006 |
| 0-4 |  | 257 (78%) | 62 (72%) |  |  | 225 (79%) | 55 (71%) |  |  | 223 (82%) | 36 (64%) |  |
| 5-15 |  | 73 (22%) | 24 (28%) |  |  | 61 (21%) | 22 (29%) |  |  | 48 (18%) | 20 (36%) |  |
| median (IQR) | 416 | 2.0 (1.0, 4.0) | 2.0 (1.3, 5.0) | 0.047 | 363 | 2.0 (1.0, 4.0) | 3.0 (2.0, 5.0) | 0.008 | 327 | 2.0 (1.0, 4.0) | 3.0 (2.0, 5.2) | 0.004 |
| Habitual Alcohol Consumption n (%) | 402 | 127 (40%) | 27 (32%) | 0.2 | 351 | 116 (42%) | 26 (35%) | 0.3 | 318 | 105 (40%) | 15 (28%) | 0.10 |
| Current smoker, n (%) | 411 | 82 (25%) | 25 (30%) | 0.3 | 358 | 76 (27%) | 24 (32%) | 0.3 | 324 | 74 (28%) | 16 (29%) | 0.9 |
| BMI in kg, n (%) | 413 |  |  | 0.034 | 361 |  |  | 0.031 | 326 |  |  | 0.11 |
| < 18,5 |  | 1 (0.3%) | 2 (2.4%) |  |  | 2 (0.7%) | 0 (0%) |  |  | 2 (0.7%) | 0 (0%) |  |
| ≥18,5 and < 25 |  | 110 (33%) | 27 (32%) |  |  | 102 (36%) | 24 (32%) |  |  | 90 (33%) | 19 (34%) |  |
| ≥25 and < 30 |  | 146 (44%) | 28 (33%) |  |  | 125 (44%) | 25 (33%) |  |  | 119 (44%) | 17 (30%) |  |
| ≥ 30 |  | 72 (22%) | 27 (32%) |  |  | 56 (20%) | 27 (36%) |  |  | 59 (22%) | 20 (36%) |  |
| median (IQR) | 413 | 27.0 (24.2, 29.4) | 27.3 (24.1, 31.3) | 0.8 | 361 | 26.8 (24.0, 29.3) | 28.0 (24.2, 31.6) | 0.030 | 326 | 26.9 (24.2, 29.4) | 27.4 (24.6, 32.0) | 0.3 |
| Cardiovascular risk factors, n (%) |  |  |  |  |  |  |  |  |  |  |  |  |
| Diabetes mellitus | 416 | 64 (19%) | 21 (24%) | 0.3 | 363 | 60 (21%) | 16 (21%) | >0.9 | 327 | 49 (18%) | 16 (29%) | 0.073 |
| History of myocardial infarction | 413 | 10 (3.0%) | 2 (2.4%) | >0.9 | 362 | 7 (2.4%) | 4 (5.3%) | 0.3 | 325 | 8 (3.0%) | 1 (1.8%) | >0.9 |
| Coronary artery disease | 416 | 41 (12%) | 21 (24%) | 0.005 | 363 | 34 (12%) | 19 (25%) | 0.005 | 327 | 29 (11%) | 13 (23%) | 0.011 |
| Peripheral artery disease | 416 | 14 (4.2%) | 8 (9.3%) | 0.10 | 363 | 15 (5.2%) | 5 (6.5%) | 0.8 | 327 | 14 (5.2%) | 3 (5.4%) | >0.9 |
| Arterial hypertension | 416 | 202 (61%) | 59 (69%) | 0.2 | 363 | 181 (63%) | 49 (64%) | >0.9 | 327 | 162 (60%) | 38 (68%) | 0.3 |
| Atrial fibrillation | 416 | 63 (19%) | 19 (22%) | 0.5 | 363 | 48 (17%) | 17 (22%) | 0.3 | 327 | 45 (17%) | 11 (20%) | 0.6 |
| Stroke subtype according to TOAST, n (%) | 416 |  |  | 0.2 | 363 |  |  | 0.5 | 327 |  |  | 0.8 |
| Large Artery Atherosclerosis |  | 88 (27%) | 25 (29%) |  |  | 74 (26%) | 23 (30%) |  |  | 70 (26%) | 16 (29%) |  |
| Cardioembolic |  | 73 (22%) | 22 (26%) |  |  | 61 (21%) | 17 (22%) |  |  | 59 (22%) | 10 (18%) |  |
| Small Artery Occlusion |  | 51 (15%) | 16 (19%) |  |  | 47 (16%) | 8 (10%) |  |  | 43 (16%) | 10 (18%) |  |
| Other cause |  | 14 (4.2%) | 0 (0%) |  |  | 8 (2.8%) | 4 (5.2%) |  |  | 8 (3.0%) | 3 (5.4%) |  |
| Undefined |  | 104 (32%) | 23 (27%) |  |  | 96 (34%) | 25 (32%) |  |  | 91 (34%) | 17 (30%) |  |
| Pre-stroke institutionalization, n (%) | 416 | 4 (1.2%) | 1 (1.2%) | >0.9 | 363 | 5 (1.7%) | 0 (0%) | 0.6 | 327 | 4 (1.5%) | 0 (0%) | >0.9 |
| ≤ 10 years Education, n (%) | 403 | 204 (64%) | 68 (80%) | 0.013 | 353 | 167 (60%) | 60 (79%) | 0.003 | 318 | 159 (61%) | 47 (84%) | <0.001 |
| MRI findings for WahlundScore^1^ | 303 | 4.0 (2.0, 8.0) | 5.0 (3.2, 8.8) | 0.4 | 259 | 5.0 (2.0, 9.0) | 4.0 (2.8, 6.2) | 0.085 | 235 | 5.0 (2.0, 8.2) | 5.0 (4.0, 8.0) | 0.4 |
| Physical activity pre-stroke, n (%) | 414 |  |  | 0.6 | 360 |  |  | 0.2 | 324 |  |  | 0.090 |
| None to occasional |  | 211 (64%) | 58 (67%) |  |  | 170 (60%) | 53 (69%) |  |  | 163 (61%) | 40 (73%) |  |
| Regular to heavy |  | 117 (36%) | 28 (33%) |  |  | 113 (40%) | 24 (31%) |  |  | 106 (39%) | 15 (27%) |  |
| MRI Infarct pattern, n (%) | 279 |  |  | 0.4 | 241 |  |  | 0.6 | 216 |  |  | 0.2 |
| Territorial infarct |  | 75 (33%) | 17 (34%) |  |  | 62 (31%) | 18 (42%) |  |  | 57 (30%) | 10 (34%) |  |
| Subcortical infarct |  | 60 (26%) | 11 (22%) |  |  | 49 (25%) | 10 (23%) |  |  | 54 (29%) | 8 (28%) |  |
| Scattered infarct |  | 47 (21%) | 15 (30%) |  |  | 43 (22%) | 8 (19%) |  |  | 36 (19%) | 9 (31%) |  |
| Lacunar Infarct |  | 0 (0%) | 0 (0%) |  |  | 0 (0%) | 0 (0%) |  |  | 0 (0%) | 0 (0%) |  |
| Infratentorial Infarct |  | 47 (21%) | 7 (14%) |  |  | 44 (22%) | 7 (16%) |  |  | 40 (21%) | 2 (6.9%) |  |
| ^1^ Wilcoxon rank sum test; Pearson's Chi-squared test; Fisher's exact test |  |  |  |  |  |  |  |  |  |  |  |  |
|  | | | | | | | | | | | | |
|  | | | | | | | | | | | | |

Supplemental Table 2. Quartiles of high sensitivity C-reactive protein levels at baseline and depressive symptoms over time in patients with ischemic stroke

|  | Crude | | Adjusted | | Adjusted  + male interaction | | Adjusted  + female interaction | |
| --- | --- | --- | --- | --- | --- | --- | --- | --- |
|  | Change in CES-D score over time | | | | | | | |
|  | Beta | 95% CI | Beta | 95%- CI | Beta | 95% CI | Beta | 95% CI |
| hs-CRP <1.77 mg/l | Ref |  | Ref |  | Ref |  | Ref |  |
| hs-CRP 1.77 – 4.77 | 1.34 | -0.50 – 3.17 | 0.44 | -1.34 – 2.23 | 0.22 | -1.96 – 2.40 | 1.33 | -1.74 – 4.40 |
| hs-CRP  4.77 – 12.5 | 1.67 | -0.17 – 3.51 | 0.77 | -1.05 – 2.59 | -0.82 | -3.03 – 1.39 | 3.87* | 0.76 – 6.99 |
| hs-CRP  12.5-101 | 3.35** | 1.47 – 5.24 | 1.96* | 0.02 – 3.91 | 1.29 | -1.13 – 3.71 | 3.53 * | 0.46 – 6.61 |

# adjusted for age, sex, physical activity pre stroke, diabetes mellitus, history of cardiovascular disease, stroke severity, BMI, regular alcohol consumption and smoking status; *p<0.05, **p<0.01, ***p<0.001

Supplemental Table 3. high sensitivity C-reactive protein and depressive symptoms over time in ischemic stroke patients with subclinical level below 10 mg/L

|  | Crude | | Adjusted | | Adjusted  + male interaction | | Adjusted  + female interaction | |
| --- | --- | --- | --- | --- | --- | --- | --- | --- |
|  | Change in CES-D score over time | | | | | | | |
|  | Beta | 95% CI | Beta | 95%- CI | Beta | 95% CI | Beta | 95% CI |
| 10log  hs-CRP | 1.53* | 0.08–2.98 | 1.20 | -0.5 –2.9 | -0.36 | -2.52 –1.79 | 3.54* | 0.91 –6.17 |

^#^ adjusted for age, sex, physical activity pre stroke, diabetes mellitus, history of cardiovascular disease, stroke severity, BMI, regular alcohol consumption and smoking status; *p<0.05, **p<0.01, ***p<0.001

Supplemental Table 4. Comprehensive Results Summary when including confounding for antidepressant and anti-inflammatory medication use

|  | Crude | | Adjusted | | Adjusted  + male interaction | | Adjusted  + female interaction | |
| --- | --- | --- | --- | --- | --- | --- | --- | --- |
|  | Change in CES-D score over time | | | | | | | |
|  | Beta | 95% CI | Beta | 95%- CI | Beta | 95% CI | Beta | 95% CI |
| Table 2.  10log hs-CRP^1^ | 1.87*** | 0.88–2.86 | 1.26* | 0.21 –2.32 | 0.63 | -0.67 –1.93 | 2.27** | 0.67 –3.88 |
| Suppl. Table 3.  10log hs-CRP^1^ | 1.53* | 0.08–2.98 | 1.06 | -0.59 –2.71 | -0.35 | -2.43 –1.73 | 3.23* | 0.96 –5.80 |
| Table 3.  10log hs-CRP^2^ | 1.67** | 0.53–2.81 | 1.41* | 0.20 –2.62 | 0.61 | -0.89 –2.11 | 2.68** | 0.81 –4.55 |

^1^ adjusted for age, sex, physical activity pre stroke, diabetes mellitus, history of cardiovascular disease, stroke severity, BMI, regular alcohol consumption and smoking status, use of antidepressant medication, use of anti-inflammatory medication

^2^ adjusted for age, sex, physical activity pre stroke, ARWMC (Wahlund Score), diabetes mellitus, history of cardiovascular disease, stroke severity, BMI, regular alcohol consumption and smoking status; use of anti-inflammatory medication, use of antidepressant medication, *p<0.05, **p<0.01, ***p<0.001
